# Increased Drought Impacts on Temperate Rainforests from Southern South America: Results of a Process-Based, Dynamic Forest Model

**DOI:** 10.1371/journal.pone.0103226

**Published:** 2014-07-28

**Authors:** Alvaro G. Gutiérrez, Juan J. Armesto, M. Francisca Díaz, Andreas Huth

**Affiliations:** 1 Department of Ecological Modeling, Helmholtz Centre for Environmental Research (UFZ), Leipzig, Germany; 2 Forest Ecology Group, Institute of Terrestrial Ecosystems, Department of Environmental Sciences, Swiss Federal Institute of Technology (ETH Zürich), Zürich, Switzerland; 3 Instituto de Ecología y Biodiversidad (IEB), Santiago, Chile; 4 Departamento de Ecología, Facultad de Ciencias Biológicas, Universidad Catolica de Chile, Santiago, Chile; 5 Departamento de Ciencias Biológicas, Facultad de Ciencias Biológicas, Universidad Andrés Bello, Santiago, Chile; Cirad, France

## Abstract

Increased droughts due to regional shifts in temperature and rainfall regimes are likely to affect forests in temperate regions in the coming decades. To assess their consequences for forest dynamics, we need predictive tools that couple hydrologic processes, soil moisture dynamics and plant productivity. Here, we developed and tested a dynamic forest model that predicts the hydrologic balance of North Patagonian rainforests on Chiloé Island, in temperate South America (42°S). The model incorporates the dynamic linkages between changing rainfall regimes, soil moisture and individual tree growth. Declining rainfall, as predicted for the study area, should mean up to 50% less summer rain by year 2100. We analysed forest responses to increased drought using the model proposed focusing on changes in evapotranspiration, soil moisture and forest structure (above-ground biomass and basal area). We compared the responses of a young stand (YS, ca. 60 years-old) and an old-growth forest (OG, >500 years-old) in the same area. Based on detailed field measurements of water fluxes, the model provides a reliable account of the hydrologic balance of these evergreen, broad-leaved rainforests. We found higher evapotranspiration in OG than YS under current climate. Increasing drought predicted for this century can reduce evapotranspiration by 15% in the OG compared to current values. Drier climate will alter forest structure, leading to decreases in above ground biomass by 27% of the current value in OG. The model presented here can be used to assess the potential impacts of climate change on forest hydrology and other threats of global change on future forests such as fragmentation, introduction of exotic tree species, and changes in fire regimes. Our study expands the applicability of forest dynamics models in remote and hitherto overlooked regions of the world, such as southern temperate rainforests.

## Introduction

Climate and forests are dynamically linked through the spatial and temporal variability of soil moisture [1], with climate system effects on ecological processes which are still poorly understood. Forest dynamics models, particularly those based on interactions among individual trees (i.e. gap models [2]), provide a simple, and general framework to assess the impacts of climate on forest dynamics. These models simulate the fate of single trees on the basis of species’ life-history traits and limited resource availability (e.g. soil moisture), thereby facilitating the analysis of climate-forest interactions [3].

Forest gap models use a variety of approaches to model forest hydrology. While some gap models use a simple bucket water balance model [3], [4], others include physiology-based representations of plant and soil controls on water uptake and evapotranspiration [5], [6]. Regardless of the level of detail used to model forest hydrology, it seems necessary that forest gap models address water availability (i.e. soil moisture) as an integrating factor, with effects on canopy transpiration [7]. Changes in rainfall regimes, summarized by changes in the duration and frequency of periods of water stress during the year, should influence soil moisture dynamics limiting plant productivity [8]. Introducing dynamic linkages of ecological processes with soil moisture variation in gap models will contribute to predict drought-induced changes in forest dynamics. Such model improvements are increasingly relevant to understanding how forests can adapt to climate change [6], [9].

Forest gap models have successfully simulated the dynamics of a variety of forest types including temperate rainforests of the southern hemisphere [10], [11]. In southern South America (SSA, 37–43°S), the progressive loss, fragmentation and subsequent degradation of temperate rainforests due to unsustainable logging and fire is threatening the integrity of ecosystem functions [12], [13] and modifying their hydrological balance [14], [15]. Annual precipitation has decreased in the same region by about 40% in the last century (time period 1901–2005, [16]) and summer rainfall is expected to decrease up to 50% by the year 2100 [17], [18]. SSA forests share similar structural characteristics with temperate rainforests of the Pacific Northwest of North America, Tasmania, and New Zealand [19]. In addition, SSA forests represent the largest area of temperate forest remaining in the southern hemisphere [20]. Floristic richness is the highest among evergreen temperate rainforests worldwide and the high concentration of endemism has given this region a unique global conservation value [20], [21]. The global relevance of SSA forests and climate trends predicted for the coming decades make it urgent to expand model applications into this region, as a tool to predict temperate rainforest responses to impending declines in rainfall.

This study introduces a forest gap model specifically designed for assessing the responses of temperate rainforests in southern South America to increased drought. The model provides accurate estimates of forest water fluxes and incorporates dynamical linkages among rainfall regimes, soil moisture, and individual tree growth. We assessed model performance by comparing the results with detailed field measurements of water cycling in a stand located on northern Chiloé Island, Chile (41°50′S). We also conducted a sensitivity analysis of the response of current forests to drought, i.e. when rainfall is decreased. Model predictions of forest hydrology (evapotranspiration and soil moisture) and structure (above-ground biomass and basal area) under increased drought predicted for 2100 in the study area were compared for a young-secondary (YS) and an old-growth (OG) forest stand to analyze differences in responses to expected changes in rainfall.

## Materials and Methods

### Study area

**Figure 1:**
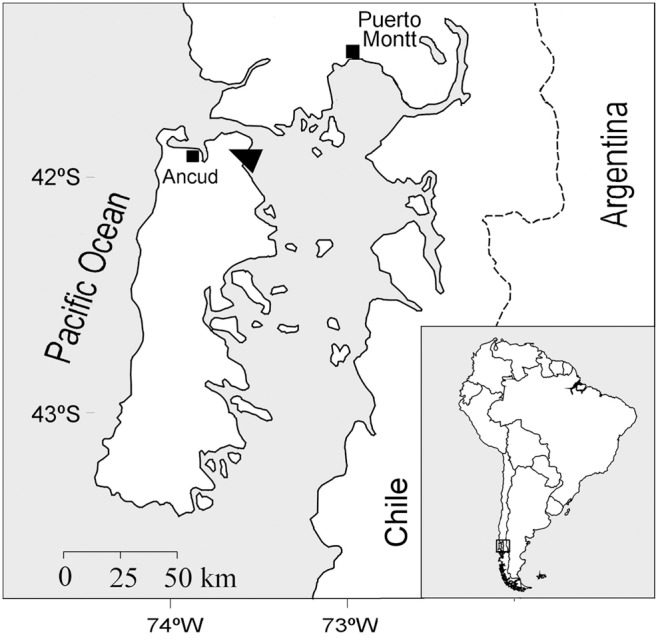
Location of study site (triangle) on northern Chiloé Island, Chile.

The study was conducted on northern Chiloé Island, Chile (41°50′ S, Fig. 1) at the private protected area *Estación Biológica Senda Darwin* (EBSD), with permission granted by the owner. Fragments of secondary and primary forests occur over rolling hills of low altitude (50–100 m) dispersed in a matrix of bogs, shrublands and grazing pastures. The present landscape has been shaped by a history of widespread use of fire to clear land for pastures since the late 1800s, followed by selective logging of remaining forest patches [22]. Soils are generally thin (<0.5 m), originated from moraine fields and outwash plains from the last glaciation, often with poor drainage [23]. Soils have high organic matter content, soil texture loam to silty loam, and a 2–4 mm thick iron silicate layer or hardpan (found at ca. 52 cm depth), where roots cannot penetrate [24]. The prevailing climate is wet-temperate with strong oceanic influence [25]. Rainfall occurs throughout the year, with an annual average of 2158 mm (25% occurring in summer). Mean annual temperature is 9.1°C. Maximum and minimum monthly temperatures are 13.9°C (January) and 4.2°C (July) [26].

Floristically, forests of the study area belong to the North Patagonian temperate rainforest type [27]. The canopy is dominated by evergreen trees, mainly *Podocarpus nubigena* (Podocarpaceae), *Drimys winteri* (Winteraceae) and *Nothofagus nitida* (Nothofagaceae), with the common presence of *Tepualia stipularis* (Myrtaceae) and several Myrtaceae tree species in the understory. Ferns (e.g. *Hymenophyllum* spp., *Hymenoglossum cruentum, Polypodium feullei)* and angiosperms (e.g. Gesneriaceae and Bromeliaceae) growing epiphytically are frequent. Detailed descriptions of structure and dynamics of this forest type have been previously published [26], [28]. The study did not involve endangered or protected tree species.

### The forest model

Here, we introduce an individual-oriented dynamic forest model (FORMIND-CL v.1.0) that includes calculations of hydrologic balance. The model is based on FORMIND, a forest model comprehensively tested to simulate the dynamics of temperate rainforests in SSA [11], [13]. FORMIND is a generalized forest growth model that simulates the spatial and temporal dynamics of uneven-aged, mixed species forest stands [29]–[31]. The model simulates forest dynamics (in annual time steps, *t*) as a mosaic of interacting forest patches of 20*×*20 m, which is the approximate crown size of a large mature tree in the forest. Within these patches, stand dynamics is driven by competition for light and space following the gap model approach [2]. For the explicit modeling of the competition for light, each patch is vertically divided into height layers of 0.5 m, where leaf area is summed up and the light environment under the canopy is calculated via a light extinction law. The carbon balance of each individual tree is modeled explicitly, including the main physiological processes (photosynthesis and respiration [13]). Allometric functions and geometrical relations are used to calculate above-ground biomass, tree height, crown diameter and stem volume from the stem diameter at 1.3 m height of the tree (*dbh*). Tree mortality can occur either through self-thinning in densely populated stands, tree senescence, gap formation by large falling trees, slow tree grow, or external disturbances (e.g. windthrow). Gap formation links neighboring forest areas. Tree regeneration rates are formulated as maximum rates of recruitment of small trees at *dbh* threshold of 1 cm, with seed loss through predation and seedling mortality being incorporated implicitly [13]. Maximum recruitment rates are reduced by shading. Nutrient availability is considered to be homogeneous at the stand scale. A description of the core model and its equations is given elsewhere [11], [13]. We focus below on the extensions added to incorporate forest hydrology.

### The hydrologic submodel

Soil moisture dynamics is described at a daily timescale, treating soil as a reservoir with an effective storage capacity that is intermittently filled by rainfall events. Soil water losses occur via transpiration, interception by the forest canopy, and drainage below the root zone. We neglected lateral water flow, thus the model applies mainly to flat terrains. This is a reasonable assumption in forests of the study area because during the rainy season soils tend to be saturated and accumulated water cannot infiltrate the soil.

Soil moisture *s* (dimensionless, 0≤ *s* ≤1), vertically averaged over the soil depth *z* (mm), was considered as central state variable [8]. Thus, the water balance equation for a given point in the forest can be expressed as [1]:
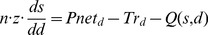
(1)where *d* is the Julian day of the year, *n* is the porosity (volume of voids/total volume of soil, i.e. dimensionless, vertically averaged); *Pnet_d_* is the net precipitation falling to the soil surface (mm day^−1^); *Tr_d_* is the transpiration rate (mm day^−1^); and *Q(s,d)* is the soil drainage (mm day^−1^). Both *n* and *z* are assumed to be time-invariant parameters [1]. The volumetric water content (*θ*, m^3^ water/m^3^ soil, i.e. dimensionless) can be calculated as follows [1]:



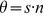
(2)The normalized version of equation (1) is used through the text where all terms are divided by *n•z*. Both the local vertical and horizontal variability of soil moisture are considered negligible at the daily timescale, assuming an equal propagation of the wetting front and equal soil moisture redistribution over the rooting zone [8], [32].

#### Net precipitation

Daily net precipitation falling to the soil surface (*Pnet_d_*) is described by,
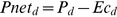
(3)where, *Ec_d_* is the canopy interception (mm day^−1^), defined here as the total daily rainfall (*P_d,_* mm day^−1^) that is retained by the canopy and is evaporated so that it does not reach the ground. Following [33], we assumed that *Ec_d_* asymptotically approaches the canopy retention capacity and can be modeled at daily intervals as:
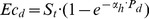
(4)where *S_t_* is the canopy water retention capacity of the stand at year *t* and *α_h_* is a parameter describing the slope of the saturation curve. The parameter *α_h_* represents, in a simplified terms, the complex process of water partitioning into throughfall and stem flow [34]. *S_t_* depends on leaf area index of the forest patch at simulated year *t* (*LAI_t_*) and is calculated by the expression [34], [35]:
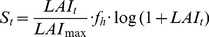
(5)where, *LAI_max_* is the maximum leaf area index of the forest and *f_h_* is a shape parameter. We avoided unrealistic canopy interception values in the model by setting *Ec_d_* = *P_d_* when *Ec_d_*>*P_d_*.

#### Soil moisture modeling

Drainage out of the root zone (*Q(s,d)*) was modeled according to [1]. When the soil is saturated (*s* = 1), soil water is permitted to percolate at a rate equivalent to the saturated hydraulic conductivity of the soil (*k_soil_*, mm day^−1^, [36]). Runoff occurs when the soil is saturated and no more water can be held in place. The excess of water is assumed to leave the system, which is reasonable to assume given the large rainfall intensity in the study area. When *s* <1, soil deep percolation rate is calculated using the empirical relationship of Neilson [37],
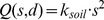
(6)


#### Transpiration

Water-use efficiency describes the proportion of water used for the assimilation of a unit of carbon in the photosynthesis [38], [39]. This concept can be used to estimate transpiration of trees (*Tr,* mmol H_2_O m^−2 ^s^−1^) from:
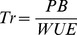
(7)where, *PB* is the gross biomass production of the tree (µmol carbon dioxide m^−2 ^s^−1^), and *WUE* is a parameter denoting water-use efficiency at stand level. *PB* is obtained from the rate of single-leaf photosynthesis following [40], which is integrated over the total LAI of the tree to account for self-shading [13]. The resulting photosynthetic rate is then multiplied by the crown area of the tree to obtain *PB* (see also equations in Appendix S1 and [13]). Daily transpiration (*Tr_d_*) of trees is obtained from equation 8 and dividing *Tr* by the length of the active photosynthetic period per year.

The daily potential evapotranspiration (*PET_d_*, mm day^−1^) describes a physical limit for the amount of water that can be held and transported away from the canopy under given climatic conditions. Evaporation is neglected in the model; therefore, it is assumed that maximum water losses by vegetation are limited by the difference between *PET_d_* and the canopy interception of the day (*Ec_d_*), as follows:
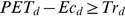
(8)
*PET_d_* is calculated using a modified Penman-Monteith expression in case of aerodynamic conductance [41], [42] and determined by the variation of the daily net radiation flux (*Rn_d_*, J m^−^^2^ day^−1^):
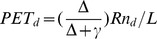
(9)where *γ* is the psychometric constant (ca. 65 Pa K^−1^, slightly depends on temperature), *L* is the latent heat of vaporization of water (ca. 2.56×10^6 ^J kg^−1^ slightly depends on temperature). The rate of change of saturated vapor pressure with temperature (Δ, Pa K^−1^) is calculated as [43], [44]:



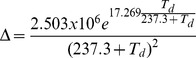
(10)
*Rn_d_* was calculated from latitude, day of the year, sunshine hours and daily air temperature (*T_d_*, °C) following [42], [45].

#### Soil moisture impact on tree biomass production

The dependence of water uptake for tree biomass production on soil moisture is described by a function representing a reduction factor due to water scarcity (*ω*(*s*), 0≤ *ω*(*s*) ≤1 [46]). This factor accounts indirectly for the impact of water demand on potential photosynthetic production (i.e. possible to achieve under competition for light). *ω*(*s*) is implemented as a daily reduction factor due to water scarcity by,
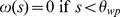


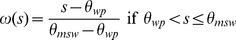


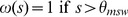
(11)where, *θ_wp_* is the wilting point, and *θ_msw_* represents a threshold when enough soil moisture is available for potential tree biomass production. We calculated *θ_msw_* from:
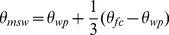
(12)where, *θ_fc_* is the soil field capacity. *θ_wp_*, *θ_msw_*, and *θ_msw_* are expressed as normalized soil moisture. In the model, the wilting point (*θ_wp_*) determines the minimum soil moisture content necessary for tree biomass production. Thus, we assumed a linear reduction of biomass production when soil water content was between *θ_msw_* and *θ_wp_*. The water required for biomass production of trees is completely removed from the soil compartment when soil moisture reaches *θ_msw_* (i.e., *ω*(*s*) = 1), after the calculation of maximum possible transpiration of trees. Both biomass production and water supply are reduced until the water needed for biomass production corresponds with *θ_wp_*. The calculated rate of biomass production influences tree respiration rate through maintenance and growth respiration, which are calculated subsequently in the model (see [13] for equations and Fig. S1 for a diagram). All calculations are performed for every tree and pooled together to calculate the stand-level values.

#### Weather generator

Rainfall time series, representing the frequency and depth of rainfall events, were constructed as series of random numbers generated by probability distributions. The interval between rainfall events, *τ* (day) can be expressed as an exponential distribution given by [47].
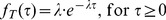
(13)where 1/*λ* is the mean time interval between rainfall events (days). Total daily rainfall (*P_d_*) depends on the amount of rain of each event (*h,* mm day^−1^), which is also assumed to be an independent random variable, expressed by an exponential probability density function [47]:
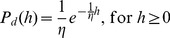
(14)where *η* is the mean depth of rainfall events (mm day^−1^). Both 1/*λ* and *η* parameters are calculated for each season of the year. We obtained daily global radiation (*Rg_d_*) from EBSD instrumental records (period from May 1998 to February 2009). *Rg_d_* varied among seasons in relation to daily rainfall (T-test, *p*<0.001). Therefore, in the model, *Rg_d_* was distributed as a Gaussian variable whose mean and standard deviation depended on *P_d_* (mean, *Rg_µ_*, and standard deviation, *Rg_σ_*). Values of *Rg_µ_* and *Rg_σ_* were obtained from instrumental records and varied depending on a threshold value of 1 mm of *P_d_* and the season of the year. Daily temperature (°C, *T_d_*) was simulated by a Gaussian random variable with parameters (mean, *T_µ_*, and standard deviation, *T_σ_*) that varied according to season of the year.

### Field data

#### Stand structure

We characterized stand structure in terms of tree species dominance (basal area, m^2^ ha^−1^) and size (*dbh*) distribution in a young secondary and an old-growth North Patagonian forest stand (hereafter YS and OG respectively) found in a flat forested area at EBSD (Fig. 1). The YS stand was initiated from a stand-replacing fire about 60 years ago and currently presented no evidence of logging. In 2007, we set up two 20×20 m plots to measure the hydrologic balance and stand structure. All trees rooted within each plot with stems >1.3 m height and >5 cm *dbh* were permanently marked with numbered aluminum tags, identified to species, and their *dbh* measured to the nearest cm. Structure and composition of YS is comparable to that described for young-secondary stands elsewhere on northern Chiloé Island (Table 1). OG was an unmanaged forest stand >590 years old, without evidence of recent human disturbance, and representative of old-growth North Patagonian forest on Chiloé Island and elsewhere in the region [26]. Sampling methods, stand history, species composition and structure of OG is described by [28]. OG had total basal area of 72 m^2^ ha^−1^ and density of 2610 trees ha^−1^, with a mixed dominance of Podocarpaceae species, *D. winteri, N. nitida* and Myrtaceous tree species in the understory [28].

**Table 1:** Structure and composition of the young-secondary North Patagonian forest stand (YS) in Senda Darwin Biological Station, compared to values reported for other secondary forest stands, elsewhere in Chiloé Island, Chile.

	Study site		Literature*				
Species	Density (trees ha^−1^)	Basal area (m^2^ ha^−1^)	Density (trees ha^−1^)		Basal area (m^2^ ha^−1^)		
*Drimys winteri*	1550	32.3	1800	4532	24.7		61.4
*Eucryphia cordifolia*	175	0.9	112	480	1.4		14.7
Myrtaceae†	1014	3.5	252	827	2.3		4.4
*Nothofagus nitida*	713	17.7	4	260	0.1		4
Podocarpaceae¥	38	0.1	80		0.5		
*Weinmannia trichosperma*	88	0.3					
Others	287.5	1.9					
Total	3575	54.8	2431	7950		46	86.9

*Source: [26], [77]–[80] only forest stands <100 years old. Only listed tree species present in YS.

† *Tepualia stipularis, Myrceugenia spp.* and *Amomyrtus spp.;*

¥ *Podocarpus nubigena, Saxegothaea conspicua*.

#### Hydrologic measurements

We estimated net precipitation using throughfall measurements taken in two YS forest plots (i.e. rainfall falling through canopy gaps plus canopy drip), adding stemflow (water running down the stems). We conducted these measurements of volume accumulated during rainfall events occurred between June 2007 and December 2010. During this time period, we also analyzed hourly records of rainfall from the meteorological station at EBSD to obtain daily incident rainfall above the canopy. Rainfall events considered in the analysis occurred with a separation of at least two hours without rain to allow for full drip from the forest canopy. Stemflow collectors consisted of a 2 mm thick smooth polycarbonate sheet molded around the stem to form a funnel. A hose led from the lowest point of the funnel to a 25 l polythene container, where the stemflow volume was collected after each rain event. Stemflow collectors were placed in 10 randomly selected trees of the two main canopy species, *D. winteri* and *N. nitida* (*dbh* >10 cm), in each plot. We eliminated two trees from our stemflow estimates that died during the study period. We converted the volume of collected water to millimeters of rain assuming that the surface of the collectors equals the projected tree crown area. Crown area was approximated by the area of an ellipse. Throughfall collectors, 0.12×2 m long (0.7 m^2^ total area per plot) gutters were held, with a slight inclination, 0.5 m above the ground at three different locations within each plot. Collectors were connected with a funnel to a 25 l polythene container. Soil matric potential was measured every 30 minutes with four sensors per plot (*WatchDog Data Loggers* 450 and 800) placed approximately in every quarter of each plot, beneath the canopy and at ca. 15 cm soil depth. Continuous soil moisture measurements were obtained for the period January 2007 to March 2009.

### Model parameterization

We used a previous model parameterization for North Patagonian forests including all main canopy tree species (11 tree species) occurring in the studied forests. The calibration, validation and robustness of this parameter set to reproduce forest stand structure is discussed in detail by [11]. Here, we describe calibration of parameters related to the inclusion of hydrologic balance into the model. New parameters needed to run FORMIND-CL v.1.0 and their values are shown in Table 2.

**Table 2:** Parameter descriptions and parameterization methods used for running simulations in FORMIND-CL v.1.0.

	Description	Value	Units	Method	Reference
**Weather generator**					
*1/λ*	Mean interval time between rainfall events¥	†	days	a	This study
*η*	Mean depth of rainfall events¥	†	mm day^−1^	a	This study
*T_µ_*	Mean daily temperature¥	†	°C	a	This study
*T_σ_*	Standard deviation daily temperature¥	†	°C	a	This study
*Rg_µ_*	Daily global radiation above canopy^¥^ ¶	†	µmol (photons)m^−2 ^s^−1^	a	This study
*Rg_σ_*	Standard deviation of Rg_µ_^ ¥^ ¶	†	µmol (photons)m^−2 ^s^−1^	a	This study
**Hydrologic submodel**					
*n*	Vertically averaged porosity of the soil	0.757	-	b	[53]
*z*	Soil depth	520	mm	b	[14]
*k_soil_*	Saturated hydraulic conductivity	4	mm day^−1^	b	[36], [54]
*α_h_*	Slope of the canopy saturation curve	0.7	-	b, c	[34]
*f_h_*	Parameter of the relationship LAI and canopystorage capacity	3	mm day^−1^	c	[35]
*LAI_max_*	Maximum LAI of the studied forest	5.5	m^2^ m^−2^	b	[81]
*WUE*	Water-use efficiency	9	g CO_2_ kg^−1^H_2_O	e	[14]
*γ*	Psychometer constant	65	Pa K^−1^	b	[43], [44]
*L*	Latent heat of vaporization of water	2.56×10^6^	J kg^−1^	e	[43], [44]
*Δ*	Rate of change of saturated vapor pressure withtemperature	c	Pa K^−1^	d	[43], [44]
*θ_wp_*	Wilting point of the soil	0.125	-	b	[36]
*θ_fc_*	Field capacity of the soil	0.3	-	b	[36]

Method refers to a: calculated from daily meteorological data from *Senda Darwin Biological Station,* period 1998–2009, b: from literature, c: calibrated with field data, d: calculated, e: calibrated using literature.

† values indicated in Table 3.

¥ per season.

¶ calculated for dry (*Pd*<1 mm) and wet days (*Pd*≥1 mm).

The parameter *f_h_* describing the relationship between leaf area index and canopy water storage capacity was calibrated following [34] and assuming that storage capacity reaches 4.9 mm day^−1^ at a leaf area index of 5.0 as measured by [14]. *LAI_max_* was set to 5.5 following the maximum value observed in other Chilean temperate rainforests [48]. The slope of the saturation curve of the canopy rain retention capacity (*α_h_)* was set according to common values for broad-leaved temperate trees [34]. To the best of our knowledge no estimation exists for water-use efficiency at stand scale in forests of the study area. Therefore we calibrated *WUE* using transpiration estimates of Díaz *et al.* [14] in Chiloé Island and the potential canopy photosynthetic rate estimated by the model for the study area under current climate (*Tr* = 296 mm year^−1^ and *PB* = 32.9 tC ha^−1^. The selected *WUE* was then confirmed by comparison with reported values from other temperate rainforests [49]–[52]. Soil characteristics (porosity and depth) followed field descriptions from Chiloé Island [53]. We set water-retention and percolation properties of the soil (parameters *θ_wp_*, *θ_fc_* and *k_soil_*) to average values [36], [54] using texture classes (loam to silty loam) described for soils in the study area [53]. Daily records of rainfall from the EBSD weather station (60 m a.s.l, period from May 1998 to February 2009) were directly used to calculate rainfall parameters for the current climate simulations (Table 3). EBSD is the nearest and most representative weather station for the climate at the study site. We calculated the mean time interval between rainfall events from the duration (days) of rain events occurring in each season. The mean depth of rainfall events was calculated by dividing seasonal rainfall sum by the amount of wet days (*P_d_* >0). These calculations were done only for seasons with >85 daily records. We avoided the potential overestimation of annual rainfall by normalizing predicted seasonal rainfall sum by prescribed seasonal rainfall averages (Table 3). The weather generator reproduced well the seasonal fluctuations in rainfall, temperature and radiation during the year with no significant departures from observed climatic records (Fig. S2, S3). Calibrated climatic parameters were assumed representative for growing conditions of North Patagonian forests on Chiloé Island and neighboring regions in the mainland.

**Table 3:** Parameter values used to run the weather generator under different climatic scenarios.

		Current climate			Future scenarios	
**Rainfall**	Seasonal sum (mm, average ± sd)	1/λ (days)	η (mm day^−1^)	Seasonal sum (mm, average)	1/λ (days)	η (mm day^−1^)
DJF	284.6±132.7	0.9	8.3	238–131	0.99–1.36	4.2–7.5
MAM	543.6±138.6	0.55	12.8	813.4	0.55	12.8
JJA	813.4±197.2	0.29	16	543.6	0.29	16
SON	424.5±144.7	0.54	9.5	382–212	0.59–0.8	4.7–8.5
Annual sum(mm, average ± sd)	2094.8±353.8					
**Temperature**	*T_µ_* (°C)	*T_σ_* (°C)			*T_µ_* (°C)	
DJF	12.5	2.6			16.5	
MAM	10.1	3.6			13.1	
JJA	8.4	4.4			10.4	
SON	9.8	3.3			11.8	
**Radiation**	*Rg_µ_* (*Rg_σ_*)	*Rg_µ_* (*Rg_σ_*)				
	*[P_d_<1 mm]*	*[P_d_*≥*1 mm]*				
DJF	1413.9 (317.0)	986.9 (378.2)				
MAM	701.2 (294.1)	398.8 (261.6)				
JJA	408.9 (159.1)	229.7 (139.4)				
SON	1065.5 (348.7)	640.8 (320.1)				

*Current climate* indicate parameters used to run the model under current climate based on instrumental records (weather station at *Senda Darwin Biological Station*, period 1998–2009). Radiation describes parameters daily global radiation *Rg_µ_* and *Rg_σ_* (the latter in brackets, µmol(photons) m^−2 ^s^−1^). Temperature is mean daily air temperature. *Future scenarios* are the range of climatic parameters that were varied to run the model under increased drought (36 scenarios in total, see *Methods* for details). *DJF:* December to February (austral summer, growing season); *MAM*: March to May (austral autumn); *JJA*: June to August (austral winter), *SON*: September to November (spring, growing season). sd: standard deviation.

### Analyses

#### Model verification

We compared field measurements of net precipitation with model predictions at daily temporal scale. For this analysis we selected 50 rain events, representing field measurement intervals <20 days long, for which accumulated rainfall during the event (i.e. sum of daily rainfall from first to last day of the event) was correctly measured by EBSD weather station, and for which <50% of containers were filled completely during the rain event. Hydrologic parameters used for model estimation of net precipitation are indicated in Table 2. LAI measurements were unavailable for YS, thus we set LAI to comparable, averaged values reported by Diaz *et al.* [14] for the same forest type. We qualitatively compared daily variation in soil moisture produced by the model with field observations. For this analysis soil matric potential obtained for an entire year was transformed to soil moisture contents using a water retention curve [55]. We set parameters of the water retention curve following [56] for loam to silty loam texture classes and particle density of the soil type under study (ca. 2.0 g/cm^3^, [53]). Model simulations were run using rainfall data for the same period of field measurements conducted over a whole year, i.e. 2008. Note that for both analyses spatial scale of model results (>1 ha) differed from the scale of field measurements (400 m^2^ plots). We also compared model results with field measurements of hydrologic balance at yearly temporal scales in temperate rainforests elsewhere in Chile (i.e. independent studies).

We tested model performance to reproduce forest structure under current climate. For this analysis, we compared tree basal areas and stand *dbh* distributions predicted by the model with measured structure of both YS and OG North Patagonian stands. Model comparison for the YS was performed after 60 years of succession, with succession initiated from a treeless state. To compare OG forest structure, we initiated simulations with stand inventory data and run the model for 1000 years to allow the simulated stand to reach dynamic equilibrium. We compared data at the end of the simulations with the known OG structure [28]. For each stands, we ran 100 simulations of 1 ha (i.e. 25×0.04 ha forest patches, 2500 patches in total) using current climate parameters and model parameters listed in Table 2 and 3. Demographic and species parameters were taken from [11] (site Tepual). To assess the consequences of the hydrologic submodel for the simulated forest composition, we compared species-specific basal areas reported by [11] with data from the simulations using the model version described in this study. Simulations were conducted following methods outlined by [11] and results were compared at the corresponding successional ages of YS and OG.

#### Simulations under increased drought

We tested the sensitivity of model predictions for forest structure and hydrological balance to changes in rainfall regimes. Climatic scenarios were selected to represent the potential range of expected climate change (here increased drought) predicted for this century in the study area. To this end, we used a regional climate model downscaled for Chilean landscapes (PRECIS-DGF model, [17]). The business-as-usual scenario provided by PRECIS-DGF suggests a 50% decrease in rainfall during the growing season by year 2100. We used the daily scale output of the PRECIS-DGF model for year 2100 to calculate seasonal climatic parameters for year 2100. We linearly interpolated this seasonal rate of change between 1998–2009 and 2100. We used this scenario to set the limit of change in rainfall by year 2100 and developed climatic scenarios covering the change from current climate to the business-as-usual scenario. Climate scenarios were developed by gradually changing current climate parameters *1/λ* and *η* until they reached the estimated value for year 2100, i.e. 50% of the current value. First, we reduced *η* (mean depth of rainfall events) multiplying current values by 0.9 to 0.5 in steps of 0.1. Then, we increased the parameter 1/*λ* (interval between events) multiplying current values by 1.1 to 1.5 in steps of 0.1. These two parameters were first varied separately (keeping the second parameter constant) and then both together. Parameter variations produced a total of 36 climate scenarios, i.e. six levels of each of the two rainfall parameters, including the current climate scenario (Table 3). In addition, we assessed the impact of warming trends on potential evapotranspiration, and its influence on other hydrologic components and forest structure. To this end, we ran an additional scenario including temperature changes expected for year 2100, with parameters *1/λ* and *η* kept at their current values (Table 3). We considered four output variables computed at yearly temporal scale: total basal area, above-ground biomass, evapotranspiration (computed as the sum of canopy transpiration and interception) and soil moisture. The latter is dynamically linked to forest processes such as annual gross biomass production of each tree (*PB*, cf. Eq. 8, see also Fig. S1). We described changes in forest structure at different successional stages based on simulations for both YS and OG forest stands. Simulations were initialized and ran under the same conditions described in section *Model verification*. We ran 30 simulations per scenario and for each forest stand. To assess the impacts of increased drought on forests we compared the differences between means of the studied variable under current climate and means of the same variable under climate change scenarios. Finally, we checked soil moisture influence on *PB* and *Tr* by running simulations under different soil moisture conditions. We ran 216 simulations, at a scale of one ha, by monotonically varying current rainfall depth parameter (*1/λ*) from 0 to 100% of the current value (Table 2). All other parameters were kept constant. We assessed results at the corresponding successional ages of YS and OG, with succession initiated from a treeless state. All statistical analyses were done in R [57].

## Results

### Model results under current climate

**Figure 2:**
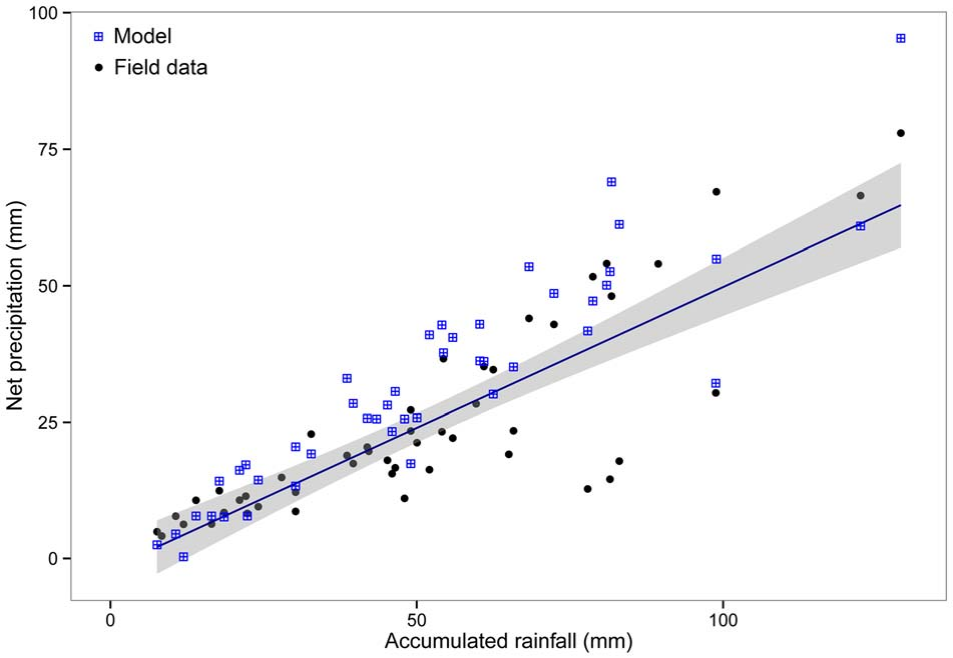
Comparison between measured and modeled net precipitation for 50 rain events recorded in a young secondary stand in northern Chiloé Island, Chile, for the time period 2007–2010. The line represents a linear regression between field measured net precipitation and accumulated rainfall during each event. The gray area represents 0.95 confidence intervals. Model results are for a forest patch are of 400^2^, with a LAI = 5.

**Figure 3:**
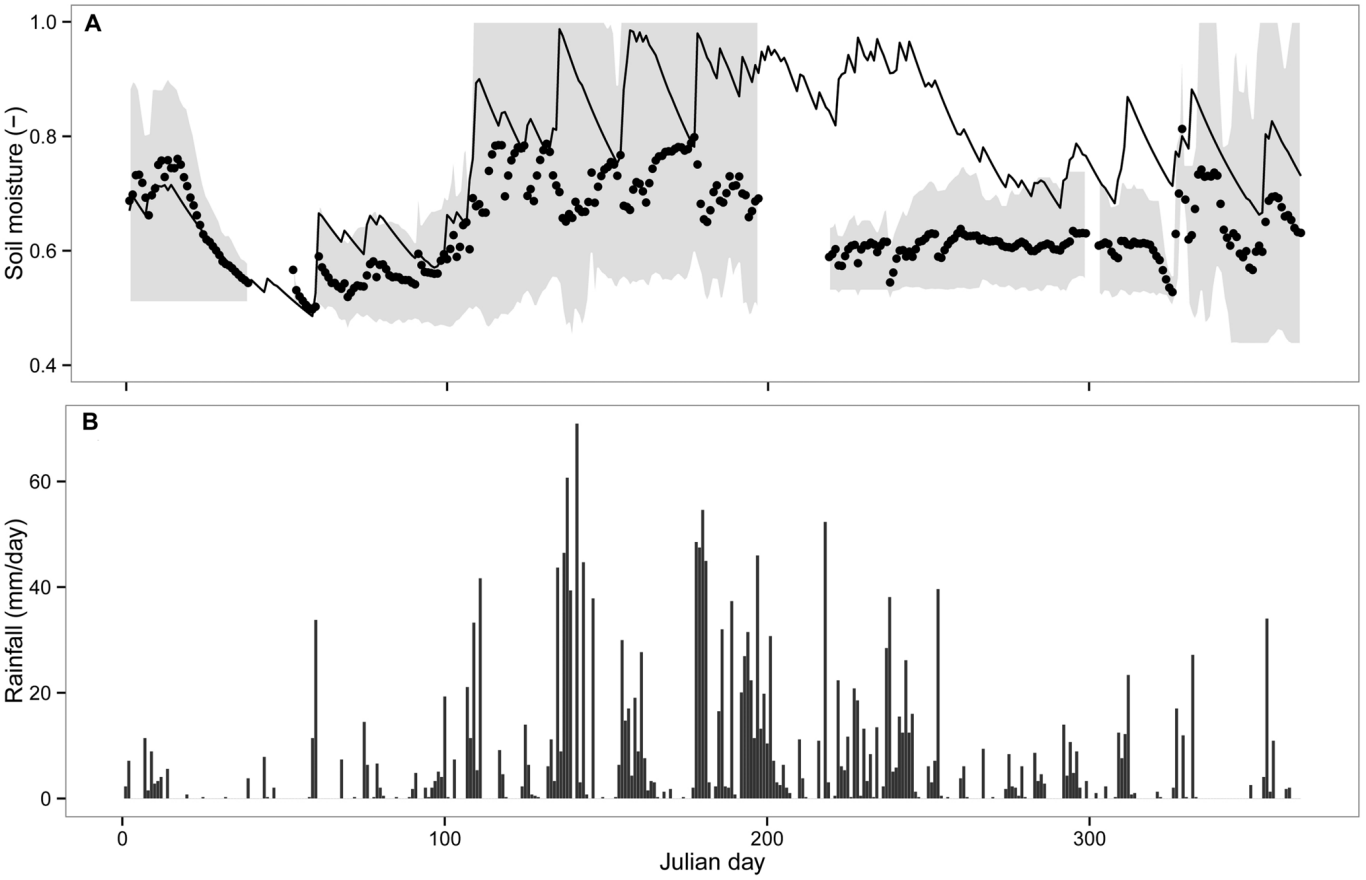
Variation in soil moisture during 2008 in a young secondary, North Patagonian forest stand (YS) in northern Chiloé Island, Chile. a) Comparison between observed and simulated soil moisture (normalized data, dimensionless). Observed data were obtained from five sensors randomly placed inside a 400-m^2^ plot in YS (daily means denoted by filled dots, gray area showing the range of data). Simulated soil moisture (line) is for one-hectare forest with a successional age of 60 years (LAI = 4.5). Soil parameters are the same as described in Table 2 (see also *Methods*). b) Rain events recorded instrumentally during 2008.

**Figure 4:**
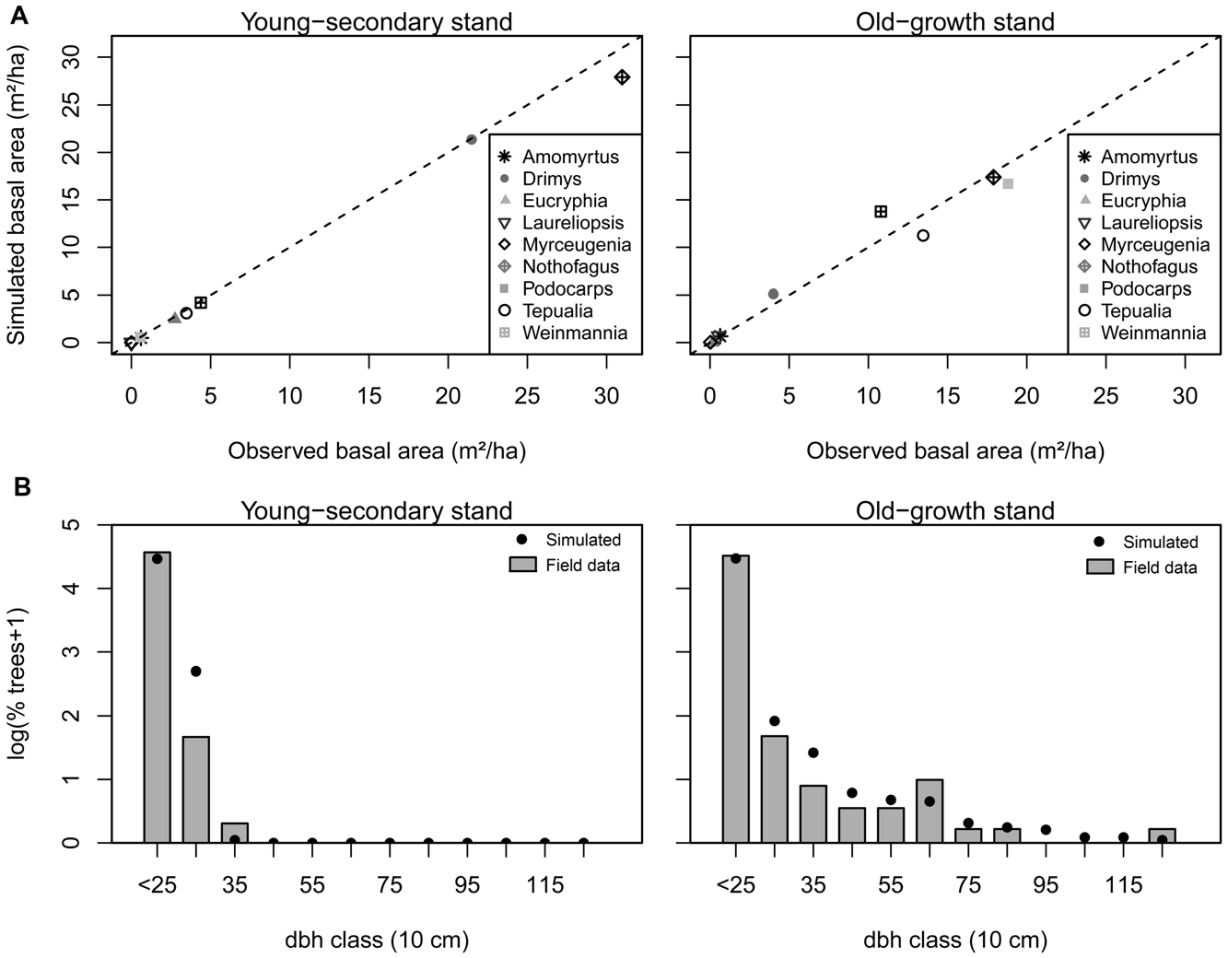
Comparison of forest structure between (a) observed (field data) and simulated basal area of tree species (Spearman’s r^2^>0.9, p<0.01 in both cases) and (b) *dbh* distributions for the young secondary (YS) and old-growth (OG) North Patagonian forest stands studied in Chiloé Island, Chile. Simulated OG structure was obtained initializing the model with inventory data.

Model predictions for the major components of forest hydrologic balance at a yearly temporal scale were similar to reported values for broad-leaved temperate rainforests in Chile and elsewhere (Table 4, independent studies). At a daily scale, the hydrologic model captured a large portion of net precipitation variability measured in the field (N = 50, r^2^ = 0.8, *P*<0.001, Fig. 2). However, for rain events accumulating >100 mm, the model predicted higher net precipitation than recorded in the field (Fig. 2). Modeled daily variation in soil moisture resembled the daily pattern of soil matric potential measured in the field during the year (Fig. 3, *r^2^* = −0.65, *p*<0.001). Soil moisture increased during austral fall and winter (Fig. 3a, 59< julian day <242) due to a higher frequency and depth of rainfall events and reduced transpiration of trees. In contrast, during the growing season, soil moisture gradually decreased according to the model and field data (Fig. 3). Forest structure simulated by the model with the inclusion of the hydrologic submodel was qualitatively similar to field data (Fig. 4a), but with some departures from observed data for small dbh-classes in YS (<35 cm, Fig. 4b). Total basal area of YS simulated by the model was similar to basal area observed in the field but total basal area of OG was underestimated (Fig. S4). However, model predictions for forest structure including the hydrologic submodel varied in the same manner across species as in the original model version and resembled field data for both stands (*r^2^* >0.97, *P*<0.001, Fig. S4).

**Table 4:** Model estimates of water balance components for a young secondary forest (YS, 60 years-old) and an old-growth North Patagonian forest (OG, >500 years-old) located in northern Chiloé Island, Chile, under current climate.

Variable	This study		Literature		
	mm year^−1^± sd	%		Value	Source*
**Young secondary stand (YS)**					
Canopy Interception	381.5±30.3	20.0		20–40%	1
Deep percolation	980.7±156.8	49.9		47%	1
ET	573.6±35.1	30.3		45.2%	1
Net precipitation	1617.2±434.6	80.0		60–80%	1
Runoff	722.9±397.7	33.3			
Soil moisture	-	62.5			
Transpiration	192.1±18.8	10.3		22%	1
**Old-growth stand (OG)**					
Canopy Interception	378.3±33.6	19.9		17.8%	2, 3
Deep percolation	907.4±142.4	46.5		66.5%	2
ET	648.0±44.1	34.3		19.9–33.3%	2
Net precipitation	1591.8±368.6	80.1			
Runoff	665.2±321.9	31.8		30–55%	4
Soil moisture	-	55.3			
Transpiration	269.7±26.1	14.4			
Potential evapotranspiration	769±4			576–724 mm year^−1^	5

Model results are the average of 100 simulations per stand (see *Methods* for details), with annual sum of rainfall averaging 1970–2000 mm year^−1^. Literature refers to values reported by independent studies in Chilean temperate rainforests and comparable forests elsewhere. sd: standard deviation; %: percentage of total annual rainfall; ET: Evapotranspiration (sum of canopy interception and transpiration). sd: standard deviation.

*(1) Data for other broad-leaved evergreen forests, ca. 200 years old. Annual rainfall 2500 mm year^−1^ [82], [83]. (2): Mixed deciduous-broad-leaved old-growth forest. Annual rainfall 2400 mm year^−1^ [12]. (3) Mixed broad-leaved and conifer forest, ca. 200 years old in New Zealand. Annual rainfall 3400 mm year^−1^ [84]. (4) Annual rainfall 1700–4500 mm year^−1^, data from evergreen, broad-leaved forests with 90% cover [85]. (5) Annual rainfall of 2427–3991 mm year^−1^ [86], weather stations of Castro and Punta Corona.

Net precipitation and runoff predictions under current climate did not differ between YS and OG stands (*P*>0.12, two-sample Wilcoxon test, Table 4). Water loss through deep percolation was lower in OG than in YS (*P*<0.001, two-sample Wilcoxon test, Table 4). Evapotranspiration was higher in OG than YS, mainly due to higher canopy transpiration rates in OG (Table 4, *P*<0.001, two-sample Wilcoxon test). Modeled soil moisture was lower in OG than in YS (0.55 and 0.63 respectively, *P*<0.001, two-sample Wilcoxon test, Table 4).

### Simulations under increased drought

**Figure 5:**
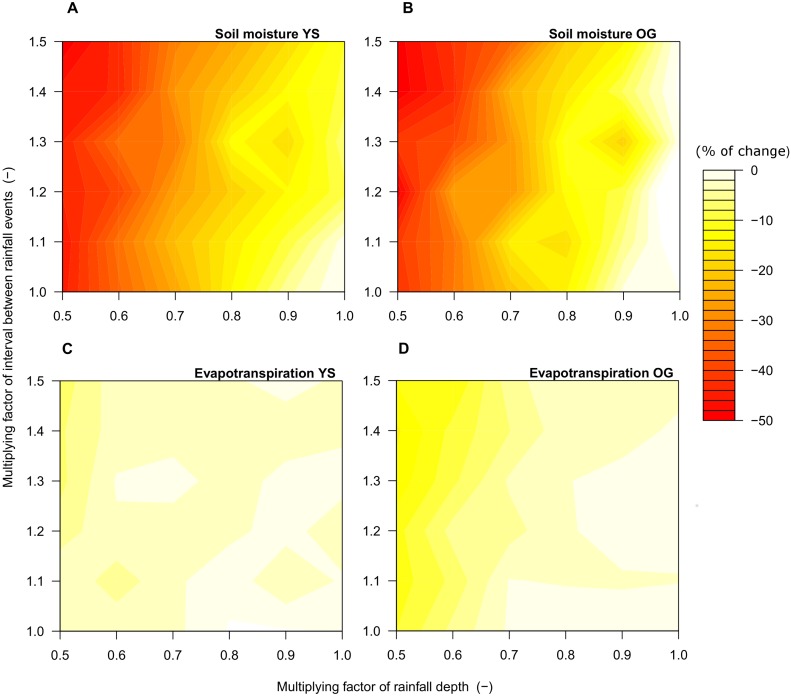
Sensitivity of soil moisture (a, b) and evapotranspiration (c, d) predicted by the model under increased drought in a young-secondary (YS) and an old-growth (OG) North Patagonian forest. Results represent the difference between the average under current climate (indicated in Table 4) and the average under future scenarios. A value of 0% indicates no change. The y-axis represents the variation of mean interval time between rainfall events (parameter 1/*λ*) and the x-axis represents the variation in the mean depth of rainfall events (*η*) under increased drought. To represent increased drought scenarios, the parameter 1/*λ* was multiplied by a factor ranging from 1 (current climate) to 1.5 whereas the parameter *η* was multiplied by a factor ranging from 1 (current climate) to 0.5. The axes of the figures correspond to these multiplying factors of rainfall parameters. Results are the averages of 30 simulations per scenario for YS and OG.

**Figure 6:**
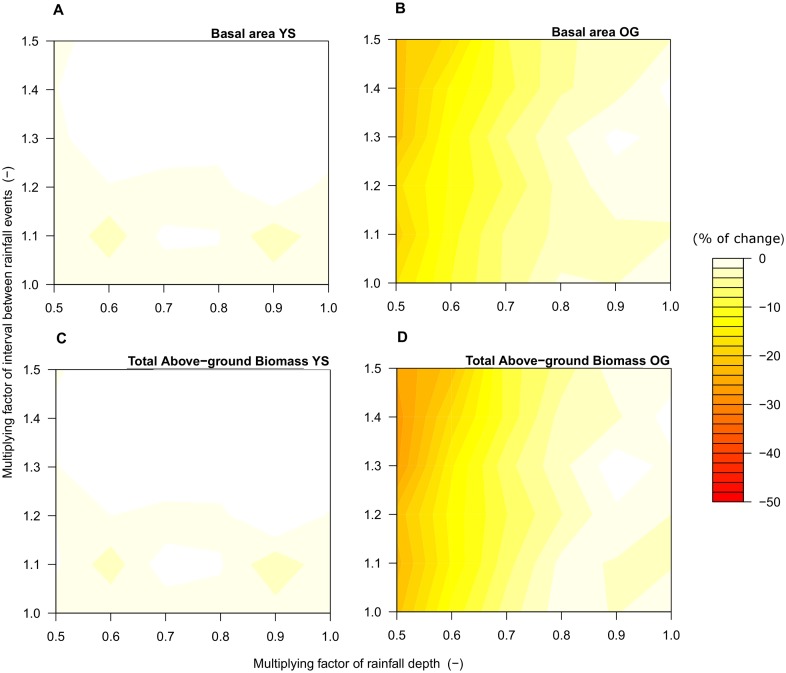
Sensitivity of total basal area (a, b) and above-ground biomass (c, d) predicted by the model under increased drought in a young-secondary (YS) and an old-growth (OG) North Patagonian forest. Results are the percentage of change between the average under current climate (indicated in Table 4) and the average under future scenarios. Results are the average of 30 simulations per scenario for YS and OG. The axes of the figures are as in Figure 5.

The model predicted changes in hydrological components and forest structure when simulations were run under expected trends of increasing drought (Fig. 5, 6). In both forest stands, YS and OG, we found similar responses of soil moisture to changes in rainfall parameters, with declines of soil moisture up to 50% (Fig. 5a, b). Reducing *η* to values <80% of the current value (Table 2, e.g. multiplying factor of 0.8) consistently reduced soil moisture by 20% (Fig. 5a, b). The influence of *1/λ* (mean interval time between rainfall events) was negligible when *η* was kept constant at its current value (Fig. 5a, b). The model consistently predicted less evapotranspiration (hereafter ET) relative to current values (Fig. 5c, d). In YS, decreases in ET under drier climate were <50 mm year^−1^ (<8% reduction relative to current values, Fig. 5c). In OG forest, ET was reduced up to 94.4 mm year^−1^ from the current value (15% reduction relative to current values Fig. 5d). Such decreases in ET occurred when current value of *η* was multiplied by 0.6 and *1/λ* was multiplied by 1.2 (Fig. 5d). An increase in PET was predicted by the model when simulations included warming trends (*P*<0.001, two-sample Wilcoxon test, Fig. S5). Only transpiration and ET in YS changed in response to warming trends and increased drought (*P*<0.05, two-sample Wilcoxon test, Fig. S5). Moreover, in both forest stands, changes in PET due to warming trends did not transfer to changes in forest structure (*P*>0.05, two-sample Wilcoxon test, see also Fig. S5).

We did not find distinct differences in basal area and aboveground biomass (AGB) in YS attributable to changes in rainfall parameters (Fig. 6). In contrast, in OG forest increased drought produced decreases in basal area by 21% of the current value (Fig. 6b, current basal area of 63.8 m^2^ ha^−1^) and decreases in AGB by 27% of the current value (Fig. 6d, current AGB of 309.5 tC ha^−1^). Main changes in basal area and AGB of OG stand were predicted when current *η* and *1/λ* parameters were multiplied by 0.6 and 1.3, respectively (Fig. 6b, d).

## Discussion

### Model performance

We developed and evaluated the performance of an individual- and process-based dynamic forest model that incorporates detailed calculations of water cycling for temperate rainforests of southern South America. The model allows for the investigation of dynamic linkages between rainfall trends and forest processes at stand scale. Parameters selected for this study were taken from the literature or calibrated using our own field data (Table 2), thus they can be considered as empirically based. The model incorporates the main known hydrological controls on forest processes in temperate rainforests of southern South America (Table 2, 4).

The accuracy of model predictions regarding forest composition and structure were comparable to previous results (Fig. S4), which were performed using a previous model version without hydrologic balance calculations [11]. Model prediction of total basal area of forests decreased with the inclusion of the hydrologic module, mainly due to fewer large trees (>100 cm dbh) predicted by the model in OG stands (Fig. 4b). However, model predictions of forest structure were considered realistic at small spatial scales (<0.2 ha) used for field sampling, compared to model results (25 ha, see also discussion in [11]). Some departures between observed and predicted daily values of net precipitation could also be attributed to canopy heterogeneity operating at different spatial scales of model results and empirical data. These discrepancies may appear when comparing forest hydrologic balance estimated for 1 ha against net precipitation measured within considerably smaller areas (<1 m^2^). Further, model predictions of daily net precipitation were based on average estimated LAI values for YS because of the lack of field measurements. LAI is a relevant variable to understanding biogeochemical cycles [58], and it should therefore be incorporated in future hydrologic analyses. Additionally, sampling errors during field measurements cannot be disregarded. Despite these limitations, we considered model predictions of forest hydrology acceptable at daily temporal scale for YS (Fig. 2, 3).

At yearly temporal scale, the model reproduced the main hydrologic components reported for similar evergreen, broad-leaved temperate rainforests in southern South America and elsewhere (Table 4, independent studies). Differences in deep percolation predicted by the present model compared to values reported in Table 4 might be accounted for specific physical characteristics of glacially originated soils on northern Chiloé Island. Deep percolation in the model is mainly depending on *k_soil_*, which was calibrated specifically for soils from northern Chiloé Island. Applying this model to forests developing on other soil types in SSA (e.g. volcanic originated soils) will require a site-specific calibration of soil parameters (Table 2). Moreover, we compared model results against a different forest type (Table 4), with shared dominance by evergreen and deciduous tree species, and differing in LAI and annual net precipitation. Despite these broad differences, Table 4 suggests that selected hydrologic parameters (e.g. *α_soil_, f_h_,* and *LAI_max_,* see Table 1) yielded reasonable values for canopy water retention capacity of broadleaved temperate rainforests in SSA.

### Forest responses to increased drought

According to predictions from our model, North Patagonian forests are likely to be altered by increased drought predicted for this century by climate change models. In our modeling study, the simulation of 50% reduction in summer rainfall predicted for the study area (business-as-usual scenario, [17]) can induce changes in both hydrological balance (up to ca. 100 mm year^−1^ decrease in ET, Fig. 5) and forest structure (up to 83 tC ha^−1^ decrease in current AGB, Fig. 6), even without considering the potential ecological effects of concomitant global warming. A direct interpretation of changes in rainfall regimes is possible because the model accounts explicitly for changes in frequency and depth of rainfall events [8], [32], [47]. Decreasing in the depth of rainfall alone can induce some structural changes in the studied forest type (Fig. 6), but simultaneous changes in the frequency and depth of rainfall produced the strongest changes in hydrology and structure of stands (Fig. 6). These results highlight the impact on forest structure and growth of the duration and frequency of water limitation periods.

**Figure 7:**
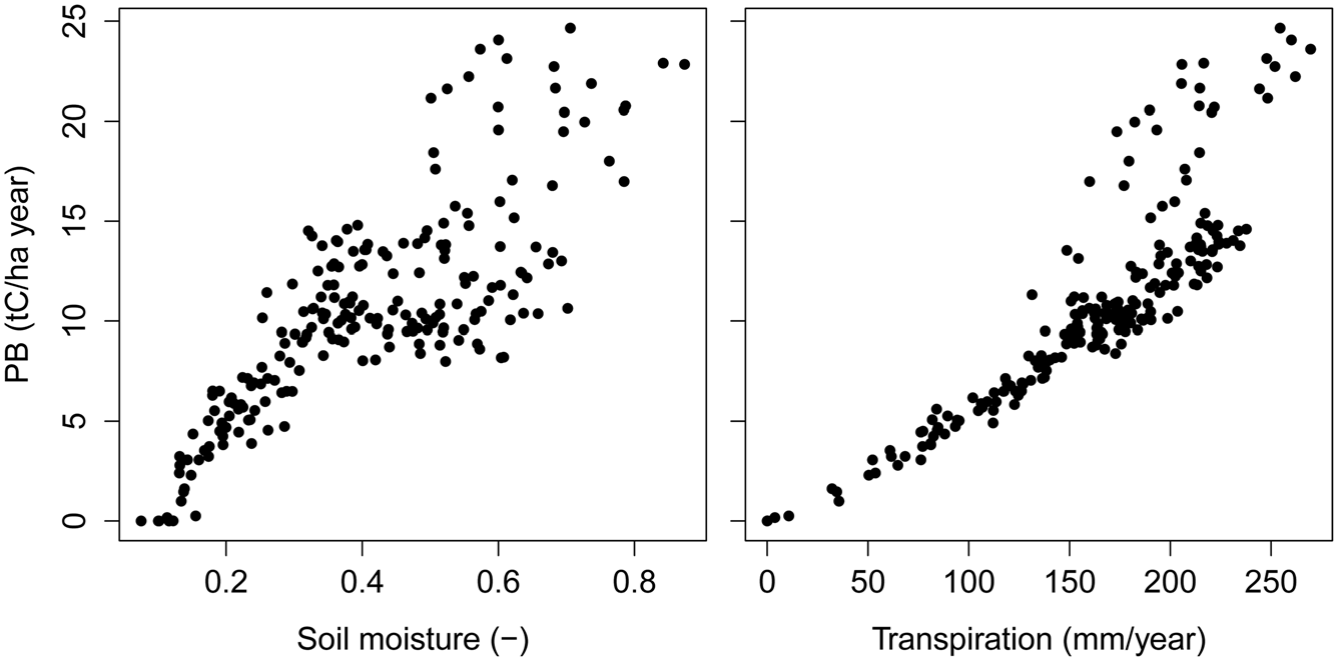
Shifts in annual gross biomass production (*PB*) of the YS North Patagonian forest stand in response to soil moisture and canopy transpiration changes in northern Chiloé Island, Chile. Results are simulations for one hectare of forest with a successional age of 60 years.

Reductions of basal area due to increased drought (Fig. 6) conform to empirical findings that drought increases the likelihood of mortality of large trees [59]–[61]. Our model formulation implies that trees assimilating greater biomass will have increasing demands of water for growth (Eq. 7, see also [62]). For example, annual gross biomass production increased with greater soil moisture availability during the year, a mechanism triggered by increased canopy transpiration (Fig. 7). Consequently, big trees that occur primarily in old-growth forests (Fig. 4b) experience increased stress-induced mortality due to greater hydrologic limitations during dry years. Trees that die under increased drought produce a decrease in both stand basal area and above-ground biomass (AGB, Fig. 6). Moreover, the model predicted that the OG forest has a higher *PB* than the young secondary stand (31.6±2.3 vs. 18.2±1.2 tC ha^−1^ year^−1^, both obtained under current climate), and consequently a higher water demand for biomass production. Under increased drought, water demand for biomass production in OG forest is not fully covered by soil moisture supply, which causes the predicted decline of AGB and basal area (Fig. 6). We propose that the contrasting ET and structural patterns between YS and OG predicted for the coming decades under increasing drought are mainly due to significant limitation of available soil moisture for biomass production in OG, with lower impact on YS.

### Research needs and model application

Here, we focused on developing an accurate model for assessing the influence of hydrologic processes involved in forest dynamics. Using the model, we quantitatively demonstrated the relative importance of soil moisture on forest structure (Fig. 6). We excluded other hydrologic processes to keep model formulation simple and results tractable with empirical information available. Processes such as water table dynamics, root dynamics, increased run off in slopes, and soil moisture dynamics across multiple soil layers can be incorporated as more empirical data becomes available for model calibration and validation. However, we strongly suggest that future model applications prioritize processes known to have an influence on the system under study [63]. In our study area, variations in the height of the water table may interfere with ecological processes such as tree establishment and mortality [14], [24]. Our model provides a convenient starting point to incorporate water table dynamics into the analysis of climate change impacts, and to explore its effects on long-term forest dynamics. Our results also highlight the need of further fieldwork and experimental research on less known, mechanistic soil parameters (e.g. *k_soil_*).

The rise of atmospheric carbon dioxide concentration and ensuing climate change are influencing water-use efficiency of forests [64], [65]. WUE under future climate is likely to differ among forests [66], local scales [67], and species [68]. Forest water-use efficiency is a sensitive parameter in our model (e.g. Fig. S6) and illustrates the need for detailed studies of the expected variations in this parameter under drought, and their connection to gas exchange capacity of trees in different topographic settings and for a large set of species in SSA. It was beyond the scope of the present study to discuss model behavior along drought-to-moist gradients operating at regional scales because such analyses require an accurate and quantitative assessment of species-specific water-use efficiency [4]. In SSA, WUE variations have been experimentally tested in few study sites and for only three tree species included in our study [49], [69]. Additional experimental research should address the complex interaction between photosynthetic carbon assimilation and water loss via transpiration with declining water supply [70]. Future research can address this question by studying changes in photosynthetic parameters along climatic gradients. Incorporating WUE as a species-specific parameter or a state dependent variable in our model is straightforward based on further empirical information.

Here, we deliberately excluded the influence of expected regional changes in temperature on forest processes (e.g. tree growth) to rather emphasize direct impacts of drought on model results. However, the model suggests that changes in PET due to expected warming trends in the study region are negligible compared to the strong impacts of increased drought (Fig. S5). Our simulated scenarios have been done using the common assumption of vegetation dynamic models that climate-forest interactions under inter-annual variation of climatic conditions can be used as proxy also for impacts of long-term climatic variations. In the later case it would be possible that some tree species would show adaptations effects. However, tree species adaptation to climate variability is still poorly understood in SSA. Moreover, warming can modify individual tree growth by affecting photosynthesis and both plant and soil respiration [38] [71], nutrient dynamics [72] and tree establishment [73]. An undergoing study analyzes the combined effect of temperature and rainfall changes on tree demography in the study area [74].

To the best of our knowledge, this is the first application of a forest gap model in temperate rainforests of SSA that integrates dynamic calculations of forest hydrology. The present work uses the best information available to ensure that climate patterns were directly comparable to hydrologic field measurements used for model calibration. However, our results on climate change impacts should be interpreted with caution because our baseline climate is constrained to a short period (1998–2009) within a long-term trend of rainfall (time period 1901–2005). Long-term monitoring of forest hydrology and dynamics can corroborate our results. To date, long-term monitoring (>10 years) of forest hydrology is lacking in forests of SSA. As more empirical data becomes available, the model can be revised and updated. The model developed here allows for the analysis of multiple environmental factors driving forest dynamics. For example, our model can help us understand the long-term responses of regional forest to drought events induced by El Niño Southern Oscillation that amplify background tree mortality rates in *Nothofagus* forest of SSA [75]. Moreover, increased regional drought is likely to interact with other drivers of global change such as changes in fire regimes, massive introduction of exotic forestry species, and forest fragmentation. To date, the implications of these drivers in the context of a changing climate remain poorly understood in SSA. These interacting growing threats demand from ecologists to understand and integrate multiple dimensions of global change on forest functioning. The model presented here is a particularly suitable tool for analyzing broad global change questions in forests of SSA because it also includes logging and fragmentation submodels [13], [76]. Model-based experiments can also contribute to develop sound management strategies that anticipate forest responses to increasing drought and other drivers of climate change.

## Conclusions

We developed and applied a forest dynamic model to analyze the impact of climate-driven increased drought on ecological and hydrological processes. The developed model was accurate for depicting forest hydrology at stand scales (i.e. <100 ha) and allowed the analysis of the dynamical linkages among rainfall regimes, soil moisture variation, and individual tree growth. Using the model we demonstrated that evergreen, broad-leaved temperate rainforests in southern South America are expected to be highly sensitive to future climate change, particularly increases in drought during parts of the year. Increased summer drought predicted for this century will likely impair biomass carbon accumulation, and amplify background tree mortality rates in this region. The developed model expands the range of applicability of gap models to assess climate change impacts in remote and understudied regions of the world, such as temperate forests of the southern hemisphere. It also represents an advance in the development of simple, general models to account for complex and dynamical processes operating at multiple spatial scales in forests.

## Supporting Information

Figure S1 **A diagram of the hydrologic submodel of **FORMIND-CL v.**1.0.** Interaction between processes and variables in the hydrologic submodel, and their respective time scales of calculations. Arrows indicate whether the results of a model calculation influence the calculations of another submodel. Blue boxes represent analyzed variables of this study. All calculations are done in yearly time steps in the model, excepting the ones indicated in the dashed box. Variable notations follow the text. AGB: Above-ground biomass, LAI: leaf area index.
(PDF)

Figure S2 **Weather generator results.** Density functions of daily rainfall and daily mean temperature predicted by the weather generator compared to observed weather records from EBSD weather station.
(PDF)

Figure S3 **Weather generator results.** Comparison between simulated and observed climatic patterns during the year. Simulations were run for 100 years using parameters in Table 3. Daily data were averaged by seasons (mean daily temperature and daily radiation). Rainfall is the amount of rainfall during each season. Observed weather data are from EBSD weather station and seasons according to table 3.
(PDF)

Figure S4 **Forest composition predictions.** Model results for forest composition using different model versions compared to field data. Simulations run under the same conditions detailed in Methods section.
(PDF)

Figure S5 **Drought induced simulations with warming included.** Changes in hydrologic components and forest structure when warming and increased drought was considered. PET: Potential evapotranspiration (mm year^−1^), T: transpiration (mm year^−1^), Ec: canopy interception (mm year^−1^), ET: evapotranspiration (mm year^−1^), BAT: total basal area (m^2^ ha^−1^), BT: Total biomass (tC ha^−1^). Result of a two-sample Wilcoxon test is shown on the upper right of each panel. Pink lines, drought induced simulations with warming included, blue lines drought induced simulations without warming, circles represent the values of simulation results. Note different scales for the axes.
(PDF)

Figure S6 **Sensitivity of evaporatranspiration.** Changes in evapotranspiration (ET) of the old-growth stand under current climate when using different water-use efficiency values (WUE). Simulations run under the same conditions detailed in Methods section.
(PDF)

Appendix S1 **Calculation of canopy photosynthetic rate in FORMIND-CL v.1.0.**
(PDF)
