# Supplementary material for: Increased Drought Impacts on Temperate Rainforests from Southern South America: Results of a Process-Based, Dynamic Forest Model
Source: PLoS One. 2014 Jul 28;9(7):e103226. doi: 10.1371/journal.pone.0103226 (PMC4113359; doi:10.1371/journal.pone.0103226)
Supplement: Figure S1 — A diagram of the hydrologic submodel of FORMIND-CL v.1.0. Interaction between processes and variables in the hydrologic submodel, and their respective time scales of calculations. Arrows indicate whether the results of a model calculation influence the calculations of another submodel. Blue boxes represent analyzed variables of this study. All calculations are done in yearly time steps in the model, excepting the ones indicated in the dashed box. Variable notations follow the text. AGB: Above-ground biomass, LAI: leaf area index. (PDF) [file pone.0103226.s001.pdf]

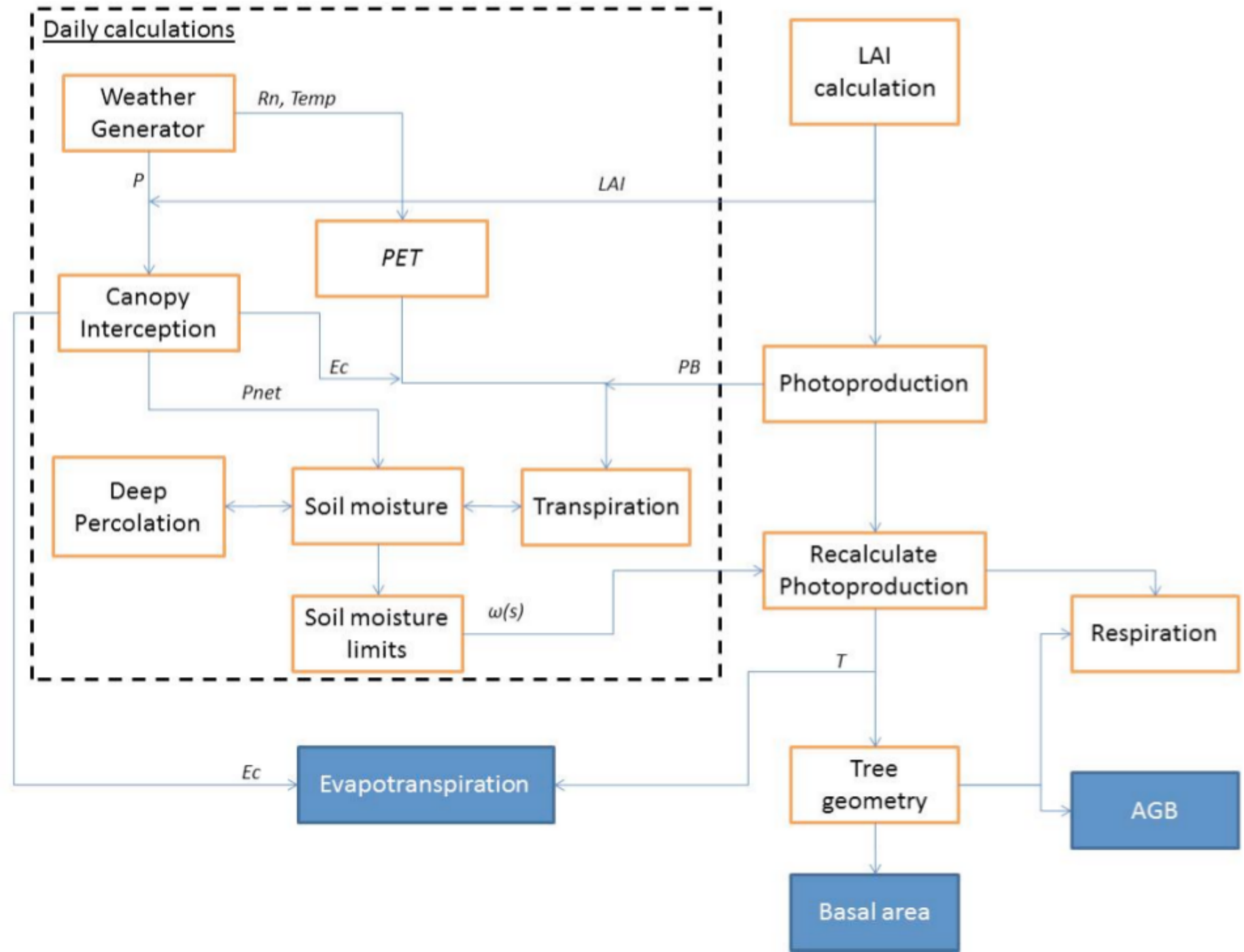
Daily calculations
Weather Generator
Rn, Temp
LAI calculation
P
LAI
PET
Canopy Interception
Ec
PB
Photoproduction
Pnet
Deep Percolation
Soil moisture
Transpiration
Recalculate Photoproduction
Soil moisture limits
ω(s)
Respiration
T
Tree geometry
Ec
Evapotranspiration
AGB
Basal area
