# Supplementary figures and images for: Increased Drought Impacts on Temperate Rainforests from Southern South America: Results of a Process-Based, Dynamic Forest Model
Source: PLoS One. 2014 Jul 28;9(7):e103226. doi: 10.1371/journal.pone.0103226 (PMC4113359; doi:10.1371/journal.pone.0103226)

**Predicted**

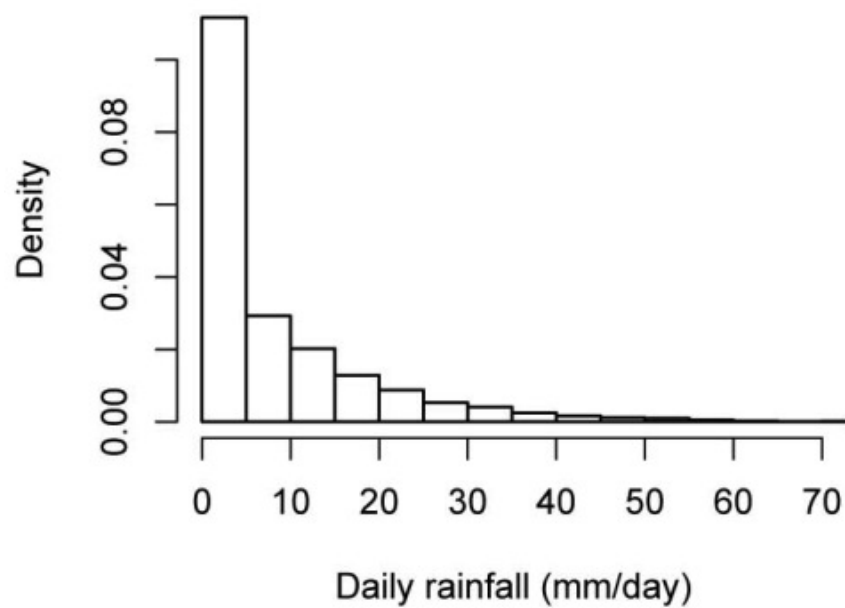

**Observed**

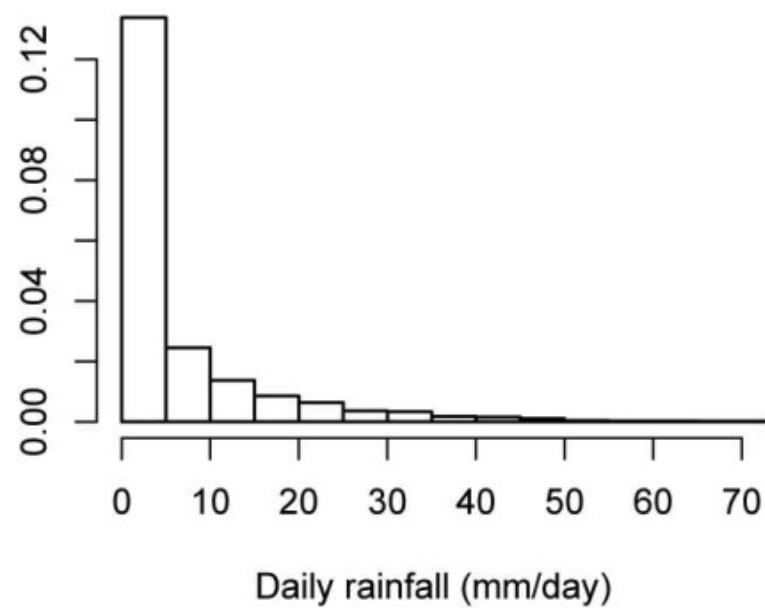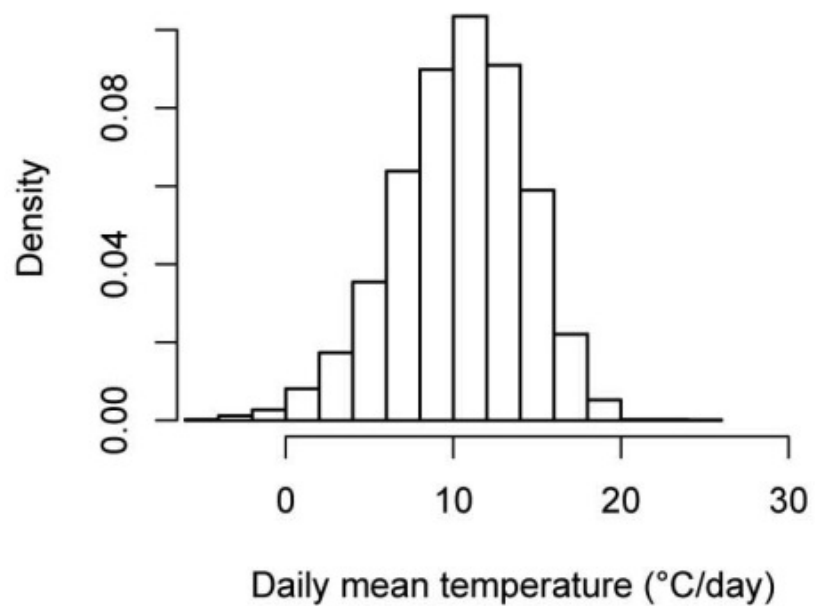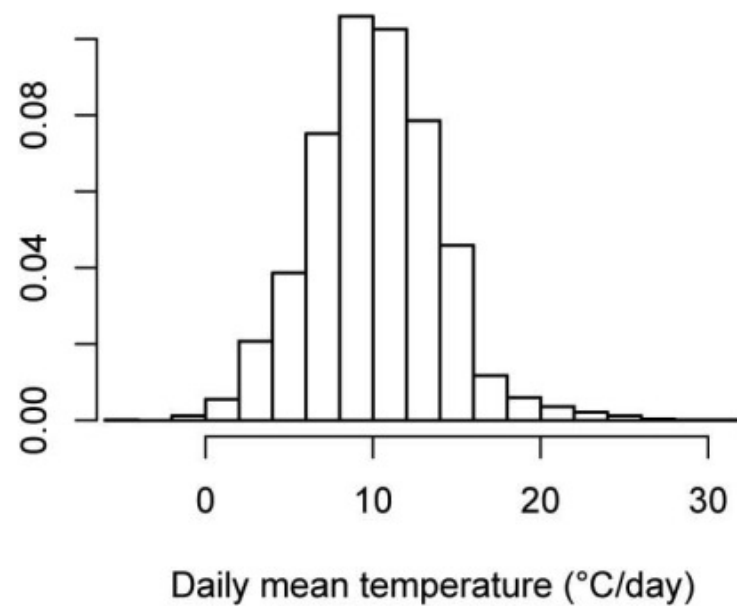

Supplement: Figure S2 — Weather generator results. Density functions of daily rainfall and daily mean temperature predicted by the weather generator compared to observed weather records from EBSD weather station. (PDF) [file pone.0103226.s002.pdf]

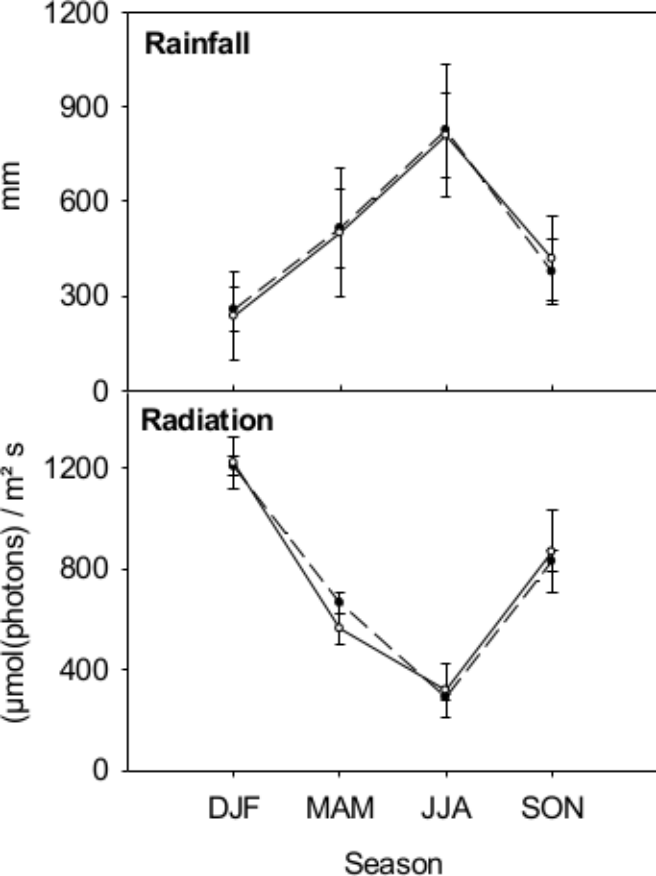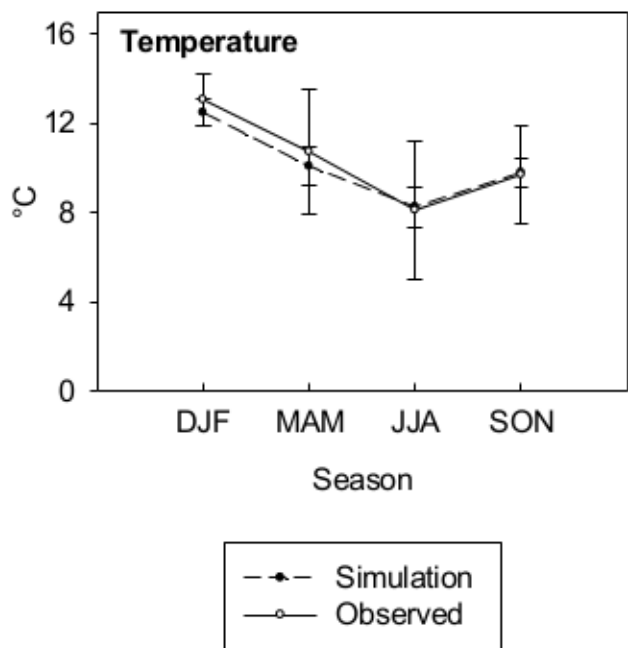

Supplement: Figure S3 — Weather generator results. Comparison between simulated and observed climatic patterns during the year. Simulations were run for 100 years using parameters in Table 3. Daily data were averaged by seasons (mean daily temperature and daily radiation). Rainfall is the amount of rainfall during each season. Observed weather data are from EBSD weather station and seasons according to table 3. (PDF) [file pone.0103226.s003.pdf]

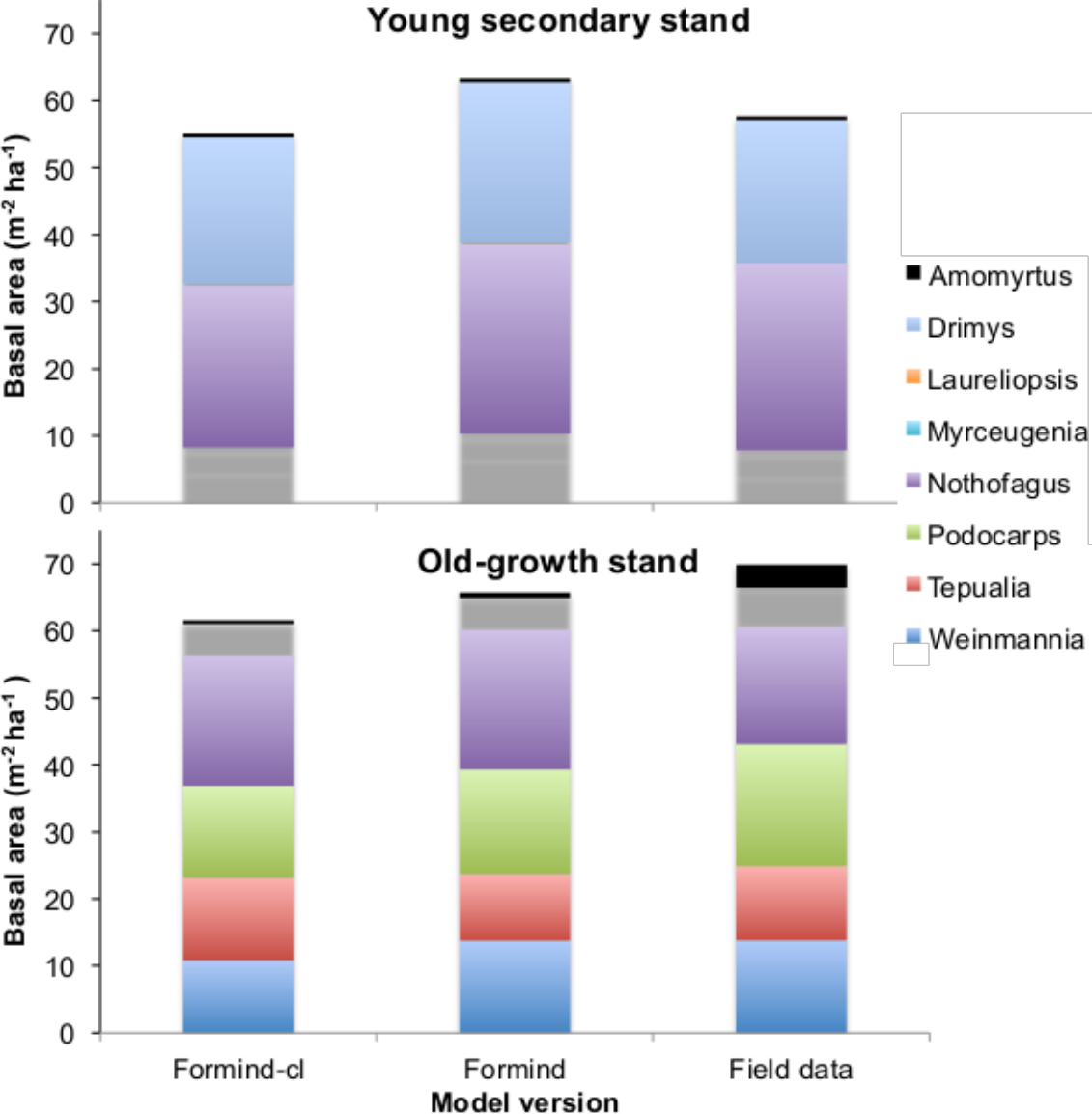

Supplement: Figure S4 — Forest composition predictions. Model results for forest composition using different model versions compared to field data. Simulations run under the same conditions detailed in Methods section. (PDF) [file pone.0103226.s004.pdf]

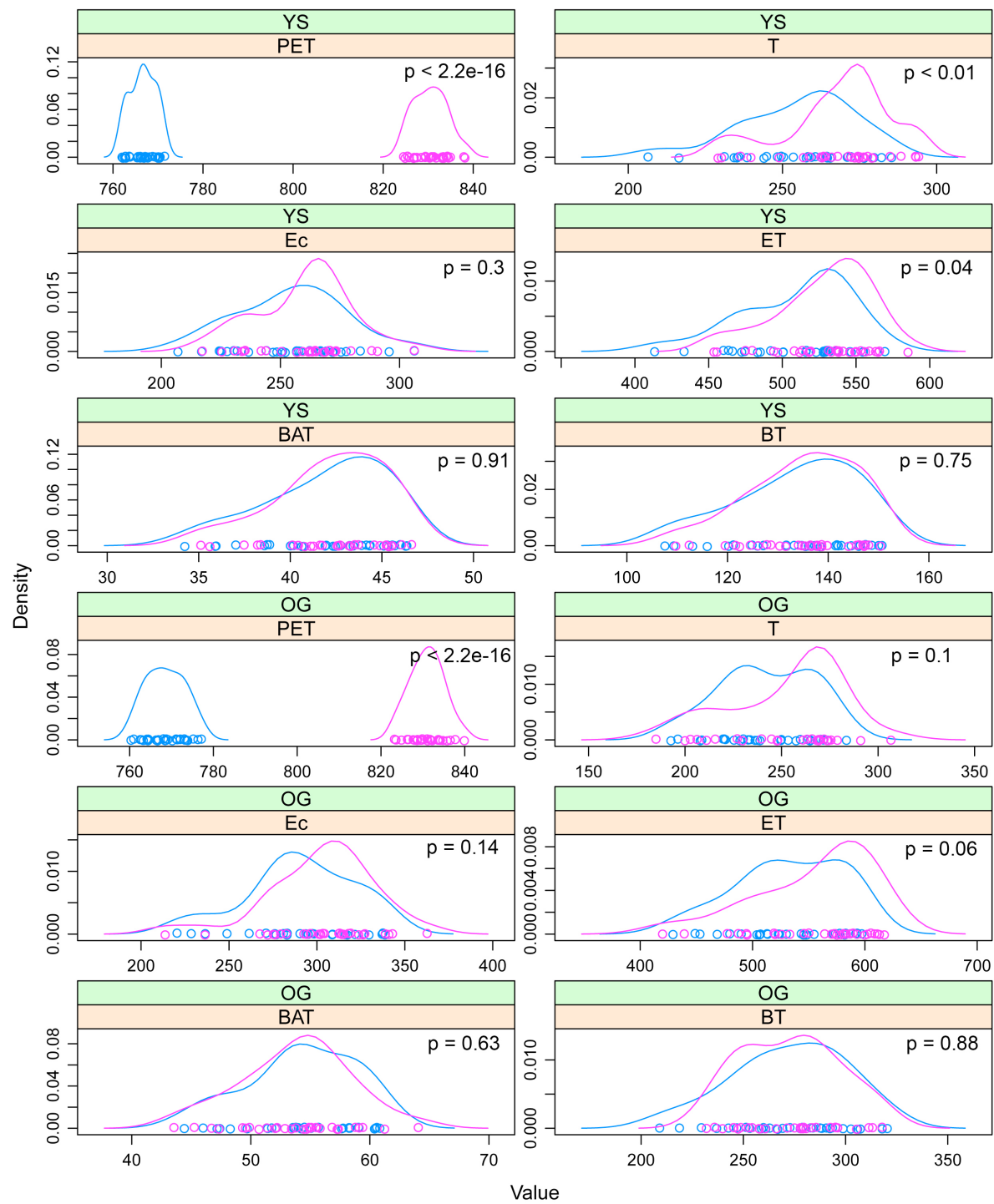

Supplement: Figure S5 — Drought induced simulations with warming included. Changes in hydrologic components and forest structure when warming and increased drought was considered. PET: Potential evapotranspiration (mm year−1), T: transpiration (mm year−1), Ec: canopy interception (mm year−1), ET: evapotranspiration (mm year−1), BAT: total basal area (m2 ha−1), BT: Total biomass (tC ha−1). Result of a two-sample Wilcoxon test is shown on the upper right of each panel. Pink lines, drought induced simulations with warming included, blue lines drought induced simulations without warming, circles represent the values of simulation results. Note different scales for the axes. (PDF) [file pone.0103226.s005.pdf]

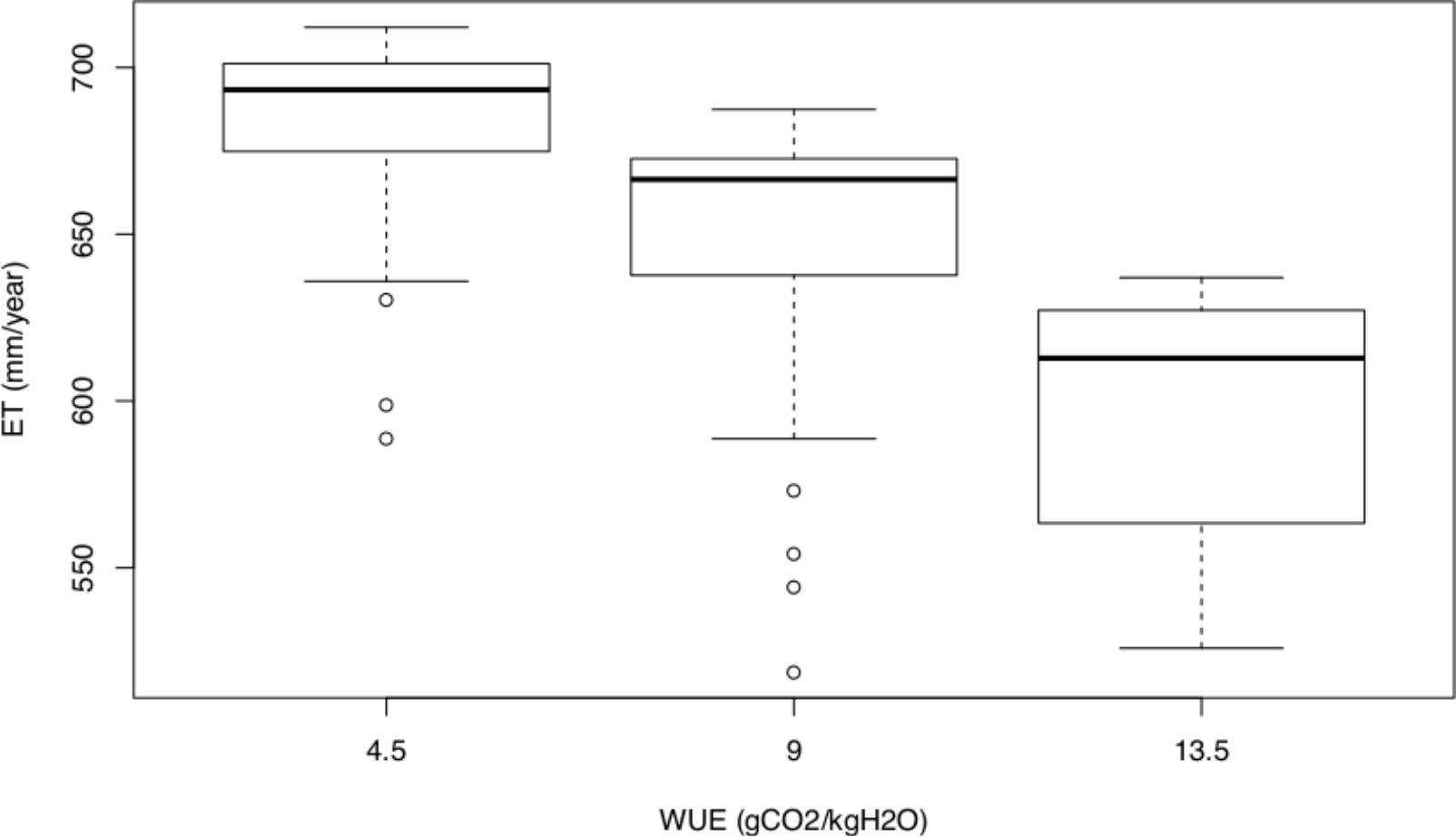

Supplement: Figure S6 — Sensitivity of evaporatranspiration. Changes in evapotranspiration (ET) of the old-growth stand under current climate when using different water-use efficiency values (WUE). Simulations run under the same conditions detailed in Methods section. (PDF) [file pone.0103226.s006.pdf]
