# Supplementary material for: Increased Drought Impacts on Temperate Rainforests from Southern South America: Results of a Process-Based, Dynamic Forest Model
Source: PLoS One. 2014 Jul 28;9(7):e103226. doi: 10.1371/journal.pone.0103226 (PMC4113359; doi:10.1371/journal.pone.0103226)
Supplement: Appendix S1 — Calculation of canopy photosynthetic rate in FORMIND-CL v.1.0. (PDF) [file pone.0103226.s007.pdf]

Appendix S1. Calculation of canopy photosynthetic rate in FORMIND-CL v.1.0

The calculation of canopy photosynthetic rate follows [1]. The rate of single-leaf photosynthesis (P_l) is a saturating function of the incident light on the leaf (I_l),

$$P_l(I_l) = \frac{\alpha I_l p_{\max}}{\alpha I_l + p_{\max}},$$

where p_{max} is the maximum rate of photosynthesis and α the initial slope of the light-response curve (both species-specific parameters). The irradiance incident on the surface of a leaf within the canopy of a tree is

$$I_l = \frac{k}{1-m} I_t,$$

with I_t being the irradiance incident on the tree crown, k the light extinction coefficient of the forest, and m the light transmission coefficient of leaves. P_l is integrated over the total LAI of tree (LAI_t) to yield the instantaneous rate of photosynthesis of the tree (P_t, per unit crown projection area),

$$P_t(I_t) = \frac{p_{\max}}{k} \ln \frac{\alpha \cdot k \cdot I_t + p_{\max}(1-m)}{\alpha \cdot k \cdot I_t \cdot e^{-k \cdot LAI_t} + p_{\max}(1-m)}$$

To calculate total annual gross biomass production of the tree (PB), the rate of photosynthesis is multiplied by the length of the photosynthetic active period per year (S), the crown area of the tree (CA) and a conversion coefficient (codm) from absorbed CO_2 to organic dry mass,

$$PB = P_t(I_t) \cdot S \cdot CA \cdot codm$$

Other equations of `FORMIND` can be found in [2] and [3].

References

1. Thornley HMJ, Johnson IR (1990) Plant and Crop Modelling – A mathematical approach to plant and crop physiology. Oxford, UK.: Clarendon Press.

2. Rüger N, Gutiérrez AG, Kissling WD, Armesto JJ, Huth A (2007) Ecological impacts of different harvesting scenarios for temperate evergreen rain forest in southern Chile - A simulation experiment. Forest Ecology and Management 252: 52-66.

3. Gutiérrez AG, Huth A (2012) Successional stages of primary temperate rainforests of Chiloé Island, Chile. Perspectives in Plant Ecology Evolution and Systematics 14: 243-256.
